# Management Practice and Clinical Outcomes of Dementia in Sub-Saharan Africa: A Systematic Review

**DOI:** 10.1155/2023/2307443

**Published:** 2023-07-25

**Authors:** Dessale Abate Beyene, Alemseged Beyene Berha

**Affiliations:** Department of Pharmacology and Clinical Pharmacy, School of Pharmacy, College of Health Sciences, Addis Ababa University, Addis Ababa, Ethiopia

## Abstract

**Background:**

Dementia is a severe neurodegenerative disorder and it is a group of acquired symptoms associated with impaired cognitive functions. In low-income settings particularly in Sub-Saharan Africa (SSA), it is often seen as part of normal aging. Environmental, behavioral, and lifestyle interventions have the potential to alter the disease course of dementia.

**Objective:**

This study is aimed to synthesize the literature/evidence(s) on the management practice and treatment outcomes of dementia in SSA.

**Method:**

Comprehensive literature was searched in PubMed database, Cochrane Library, and Google Scholar. Eligibility has been set, and based on the criteria, initially, a total of 442 results were obtained, and from those around 183 articles were duplicated. After examining titles and abstracts of records 26 articles were identified. Finally, five randomized clinical trials (RCT) and three prospective cohort studies that were reported on the management practice and treatment outcome of dementia in SSA were eligible for analysis. RCT and prospective cohort studies were used to strengthen the quality of evidence. The quality of the included RCT studies was assessed by using the Cochrane Risk of Bias Tool.

**Result:**

A total of 2781 patient data were included in the final analysis. Of these, 2354 patients were obtained from 5 RCTs and 427 patients from 3 prospective cohort studies, which were conducted in SSA countries. RCT studies were done on the feasibility and clinical effectiveness of cognitive stimulation therapy (CST) showed improvements in language memory domains and physical health. In addition, studies that focus on the management of human immunodeficiency virus-associated dementia (HIVAD) were reported to improve neurocognitively.

**Conclusion:**

CST is applicable in low-resource settings and it shows improvements in cognitive function and quality of life. Early initiation of combination antiretroviral therapy in resource-limited settings has been associated with improvement in the cognitive function of HIVAD.

## 1. Background

Dementia is a severe neurodegenerative disorder and it is a group of acquired symptoms associated with impaired cognitive functions in memory, language, and goal-directed behaviors [1–3]. It reduces a person's ability to perform everyday activities and quality of life [4]. About 90% of patients with dementia are affected by behavioral and psychological symptoms of dementia during the illness [5]. Alzheimer's disease is the most common type of dementia followed by vascular dementia and dementia with Lewy bodies. Frontotemporal degeneration and dementias associated with brain injury, infections, and alcohol abuse are less common [6, 7]. Human immunodeficiency virus-associated dementia (HIVAD) is a syndrome of progressive deterioration of memory, cognition, behavior, and motor function in HIV infected individuals during the late stages of the disease when immunodeficiency is severe [8, 9]. Subgrouping patients into populations with large vessel disease versus small vessel disease may help to advance in diagnostic specificity and may provide a way to test the efficacy of proposed therapeutic agents and treatment strategies [10].

An estimated 50 million individuals in the world are living with dementia and HIVAD may affect 30%–50% of people living with HIV [11]. It becomes a global challenge especially since the burden of disease often is greater in Sub-Saharan Africa (SSA); due to the healthcare services, coverage can be limited [12, 13]. People with dementia are usually older than 65 years with comorbidities and it is difficult to assess, and treat and may not be accurate providers of medical history for the caregivers [7, 14]. Older peoples were at high risk to die from dementia and all professionals working in end-of-life care need to make this knowledge a central part of their planning and communication [7]. The treatment outcomes for people with dementia are often poor with substantial disability and high caregiver burden [13].

Currently, there is no curative treatment exists for dementia. The discovery of new pharmacological treatments for dementia has been largely unsuccessful [15]. In the United States, guideline-based disease management programs for dementia led to substantial improvements in the quality of care for patients with dementia [16]. Environmental, behavioral, and lifestyle interventions have the potential to alter the disease course of dementia [17, 18]. Many clinical and cognitive interventions are aimed at improving the quality of life of those who are affected by the medical condition and their interventions have been developed and progressed [7].

Cognitive stimulation therapy (CST) is an intervention for people with dementia that offers a range of enjoyable activities providing general stimulation for thinking, concentration, and memory usually in a social setting [19]. CST had substantial improvements in cognition and quality of life in studies from high-income countries, equivalent to those achieved by pharmacological treatments [20]. It is preferable in Africa, due to it requires little specialist equipment and can be delivered by non-specialist health workers [13]. Psychosocial interventions are proposed to reduce the burden of disease, particularly if introduced during the early stages of dementia [21, 22].

In SSA over two-thirds of HIV patients with advanced disease had abnormal neurologic findings due to late clinical presentation, advanced levels of immunosuppression, and a high burden of frequently undiagnosed concurrent systemic infections and difficulties in diagnosis and treatment [23]. There are many strategies aimed at reducing their high burden, morbidity, and mortality including early HIV diagnosis and combination antiretroviral therapy (cART), screening and chemoprophylaxis of main opportunistic infections, improved clinical diagnosis, and management, and program strengthening [24, 25].

In low-income settings particularly in SSA, dementia is often seen as part of normal ageing in many countries and not perceived as requiring medical care [26, 27]. These factors make an evaluation, treatment, and research on dementia in these settings uniquely challenging, with specialist and culturally specific tools, and methods for assessment and monitoring of treatment required [28, 29]. This systematic review may be indicated the level and extent of dementia management strategies and treatment outcomes. On the other hand, it may indicate the treatment gap that is observed in Africa. Hence, this study aims to synthesize the literature/evidence(s) on the management practice and treatment outcomes of dementia in SSA.

## 2. Methods and Materials

### 2.1. Information Sources and Searching

This review was described by the Preferred Reporting Items for Systematic Reviews and Meta-Analysis (PRISMA) framework. A systematic search of the literature was conducted in the PubMed database, Cochrane Library, and Google Scholar from September 11th to September 30th, 2021 for a total of 20 days. The following key terms were used as text and Medical Subject Heading MeSH) terms along with the Boolean operators (“OR, AND”): first search in the electronic database using the MeSH terms with the combinations of OR; “(therapy management OR disease management), ((clinical outcomes) OR treatment efficacy/effectiveness) OR cognitive stimulation therapy.” Second, search all the above queries by combinations of AND. The result of the final search with Boolean operators was “(((((clinical outcomes) OR treatment efficacy/effectiveness) OR cognitive stimulation therapy)) AND dementia) AND ((therapy management) OR disease management). In this review, all published randomized control trials and prospective cohort studies that were reported on the management practice and treatment outcome of dementia in SSA were included.

### 2.2. Eligibility Criteria

Randomized clinical trials (RCTs) and prospective cohort studies were used to strengthen the quality of evidence. The title and abstract of the records retrieved from the medical literature were screened independently, and data were grouped according to the type of method design. Studies written in a non-English language, conference abstracts, case reports, case series, studies with limited information and unpublished documents, and also articles which are published before 2000 were excluded from this review.

### 2.3. Search Strategy

Initially, 442 articles were identified through a systematic search from PubMed database, Cochrane Library, and Google Scholar. Of those, around 183 articles are duplicates. After examining titles and abstracts of records 26 articles were identified. Out of these articles, ten of them were cross-sectional studies, three of them were mixed methods (cross-sectional + control), two of them were accepted manuscripts, two were qualitative studies, and one done on acute neurologic complications were excluded from the final analysis. Finally, only five randomized control trials and three prospective cohort studies articles were analyzed.

### 2.4. Data Extraction

The titles and abstracts of identified studies were screened and assessed the full text of potentially eligible studies. The method of data extraction from reports was done independently from the selected articles. Any controversy was resolved by consensus.

### 2.5. Key Outcomes

The main key outcome of interest was the management practice and treatment outcomes of dementia in SSA.

### 2.6. Definition of Terms

*Dementia*: a syndrome characterized by a progressive deficit in cognitive function, with a focus on memory loss and impaired social and occupational activities [3].

*CST*: a psychosocial, group-based intervention for the treatment of dementia that provides a series of enjoyable activities that stimulate thinking, concentration, and memory in general, usually in a social setting [20].

Human Immunodeficiency Virus Associated Dementia (HIVAD): if one of the following criteria is fulfilled in HIV patients [9, 30].

- No evidence of another preexisting cause for dementia [i.e., central nervous system (CNS) infections, CNS neoplasm, and cerebrovascular disease].
- Marked interference in activities of daily living.
- Marked cognitive impairment involving at least two cognitive domains by a performance of less than two standard deviations below the mean of standardized neuropsychological tests, especially in the learning of new information, slowed information processing, and defective attention or concentration.

## 3. Results

### 3.1. Study Selection

**Figure 1:**
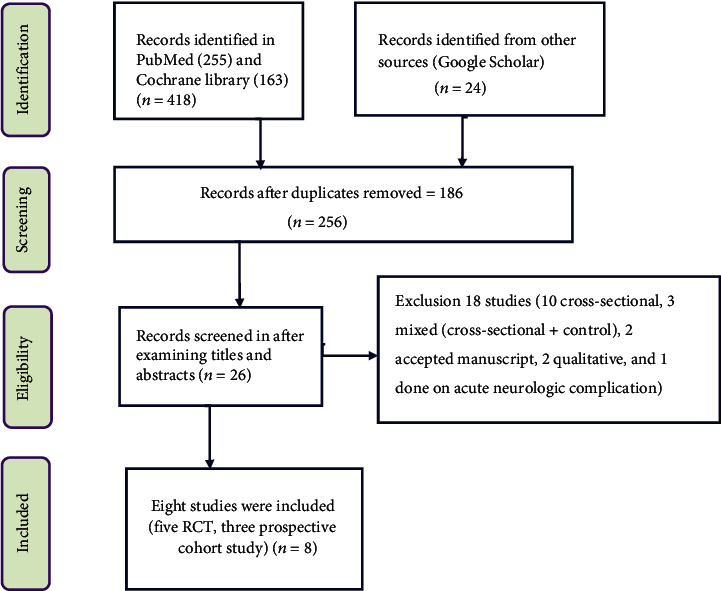
PRISMA flow chart of study selection for a systematic review of management practice and clinical outcomes of dementia in Sub-Saharan Africa, 2021.

Initially, 442 articles were identified through a systematic search from the PubMed database, Cochrane Library, and Google Scholar. Of those around 183 articles were duplicated. After examining titles and abstracts of records 26 articles were identified. Finally, five RCT and three prospective cohort studies were included in the systematic review (Figure 1).

### 3.2. Quality Assessment and Risk of Bias

**Figure 2:**
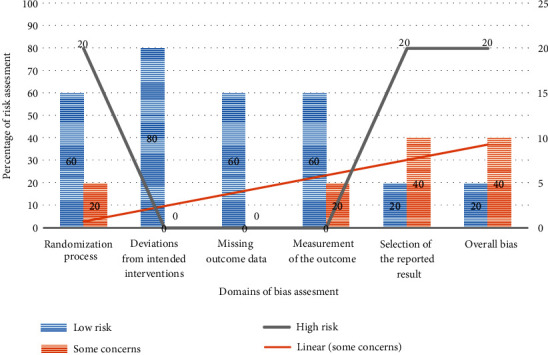
Risk of bias of RCTs included in a systematic review, 2021.

The author assessed the quality of the included RCT studies using the Cochrane Risk of Bias Tool for randomized trials (RoB 2) [13, 31–34]. It is structured into six sets of biases that focus on different aspects of study design, implementation, and reporting. Domain 1 focused on the randomization process of the intervention group, domain 2 on deviations from the intended interventions, domain 3 on missing outcome data, domain 4 on outcome measurement, domain 5 on the selection of reported outcomes, and domain 6 on overall article bias. A suggested judgment of risk of bias resulting from each domain is generated by an algorithm based on responses to signaling questions (yes, probably yes, probably no, no, and no information; Figure 2).

### 3.3. Study Characteristics

In this systematic review, five controlled clinical trial studies with 2,354 patients and three prospective cohort studies with 427 patients conducted in SSA countries published from 2006 to 2018 with 7–96 weeks of the study period were included in the final analysis [13, 31–37]. These two RCTs are done in Nigeria and Tanzania were assess the feasibility and clinical effectiveness of CST in people with dementia by using the World Health Organization Quality of Life Instruments (WHOQOL-BREF tool) [13, 34]. One RCT done in South Africa assessed the diagnostic accuracy of the international HIV dementia scale-HIV cognitive symptom questionnaire **(**IHDS-HCSQ with HIVAD) and one RCT done in five SSA countries compared with the neurocognitive efficacy of second-line cART after the first-line cART failure [32, 33].

RCT done in Uganda assessed the efficacy and safety of minocycline in the management of HIV-associated cognitive impairment and the other interventional cohort study done in Nigeria and Tanzania identified intervene dementia in elderly Africans (>65 years) by using the identification and intervention for dementia in elderly Africans (IDEA) cognitive screening tool [34, 35]. Two cohort studies done in Uganda have assessed the benefits and risks of stavudine-based cART for HIV-associated cognitive impairment and the neuropsychological test and functional performance of cART in HIV individuals, respectively (Table 1) [36, 37]. Due to the heterogeneity of study design, data collection technique, and data presentation, we did not generate summary data in the systematic review.

### 3.4. CST for Dementia

Of the included published articles, only two of them assessed the effectiveness of CST for the management of dementia in SSA. The two articles reported that CST intervention was feasible and it has the potential to be low-cost, sustainable, and adaptable to other settings across SSA [13, 31]. RCTs have been done in rural Nigeria on the feasibility and clinical effectiveness of CST post-treatment cognitive scores showed improvement in cognitive function, quality of life (physical, psychosocial, and environmental domains), physical function, neuropsychiatric symptoms, and career burden [31]. In other articles done in Tanzania on the feasibility and clinical effectiveness of CST, mild or moderate dementia participants were allocated to four CST groups and there were substantial improvements in cognition, anxiety, and behavioral symptoms after the implementation of CST [13].

### 3.5. HIV-Associated Dementia Management in SSA

The included studies focus on the management of HIVAD in SSA. A study done in South Africa focused on the diagnostic accuracy of HCSQ with or without HCSQ; the IHDS-HCSQ combination offers a viable and quick way to screen people living with HIV for HAD. It can deliver excellent sensitivity and good specificity, is easy to administer, time and cost-efficient in the assessment of HIVAD in SSA [32]. RCT study was done in five SSA countries on the administration of second-line therapy of cART for patients who failed first-line cART. Patients in SSA failing the first-line cART regiment had low neurocognitive function test scores, but performance improved on the second-line cART irrespective of the cART regimens used in the study [33].

A prospective cohort study was done in Nigeria and Tanzania both in the outpatient and inpatient setting on the validation IDEA cognitive screen tool for SSA people. IDEA cognitive screen performed well in all three settings and it can be administered by non-specialist healthcare workers with an Inter-rater reliability correlation coefficient of 0.742–0.791 and inter-rater reliability correlation coefficient greater than or equal to 0.75, corresponding to good reliability [35]. the cART can be associated with improvement in neurocognitive and functional performance in HIV individuals in SSA. One prospective cohort study done in Uganda shows improvement in the neurocognitive and functional performance of HIVAD individuals after the administration of cART. The provision of cART in areas with limited resources like SSA was also associated with improvement in functional performance [37]. Another prospective cohort study done in Uganda on HIV individuals with cognitive impairment improve significantly as demonstrated by improved performance on a test of executive function after the initiation of stavudine-based cART [36].

### 3.6. Minocycline Treatment for Dementia

Only one RCT is available in SSA on the safety and efficacy of minocycline for dementia people, the drug was safe and well-tolerated in HIV-positive individuals, but it had no significant effect over placebo in the improvement of cognitive function in cART-naive, HIV-positive patients [34].

## 4. Discussion

This systematic review aimed to offer a synthesis of existing management practices and treatment outcomes of dementia in SSA. A total of five RCT studies with 2354 patients and three prospective cohort studies with 427 patients done in SSA countries were retrieved and analyzed [13, 31–37]. The treatment outcomes for people with dementia in resource-limiting settings like SSA were often poor. People with dementia are usually older than 65 years with comorbidities and this can be making the assessment of management practice of dementia in SSA difficult for accurate providers of medical history to the caregivers [7, 13, 14].

Psychosocial interventions are proposed to reduce the burden of disease, particularly if introduced during the early stages of dementia [21, 22]. RCT has been done in Nigeria rural area patients after 7 weeks of CST intervention the cognitive scores show improvement in language and memory domains [31]. RCT done in Tanzania on the effects of CST intervention after 20 weeks of treatment in people living with dementia was show changes in quality of life scale like in cognition and the intervention has the potential to be low-cost, sustainable, and adaptable to other settings across SSA [13]. The effectiveness of CST intervention for dementia is also confirmed in high-income settings. A study done in the United Kingdom indicated that a home-based version of CST intervention showed good outcomes for people with dementia having limited access to community mental health [38], and another pilot study of longer-term maintenance CST done in the United Kingdom found a significant improvement in cognitive function [39]. Another single-blind RCT done on people with dementia in Japan indicates that CST was shown improvements in cognition on neurobehavioral cognitive status examination and mini-mental state examination [40]. This output indicates that CST can be an effective treatment option in SSA countries for dementia patients, due to it requires little specialist equipment, is easily implemented in different setups of the healthcare system like in limited healthcare services and it can be delivered by non-specialist health workers.

HIVAD was common in SSA [41]. In developing nations, most patients had a late clinical presentation of HIV, advanced levels of immunosuppression and a high burden of frequently undiagnosed concurrent systemic infections, and difficulties in diagnosis and treatment of HAND [23]. RCT done in South Africa on the diagnostics accuracy of dementia, IHDS-HCSQ combination offers a viable and quick way to screen people living with HIV for HIVAD and it can deliver excellent sensitivity and good specificity [32]. The other cohort study in Nigeria, Tanzania shows that the IDEA cognitive screen performed well in these populations and should prove useful in screening for dementia and delirium in other areas of SSA [35]. The availability of validated screening tools for dementia in resource-limiting settings may help to screen the patients early and it makes the treatment outcomes good.

A study done in Uganda indicates that patients in SSA failing the first-line cART had low neurocognitive function test scores, but performance improved on the second-line cART [33]. This might be due to the available first-line cART in most SSA countries containing efavirenz which has neurotoxicity as a major drawback [42]. On the other hand, studies indicated that there is no evidence of the neurocognitive benefit of second-line cART due to their CNS penetration capacity in HIVAD [33, 43]. A prospective cohort study was done in Uganda on the effectiveness of stavudine-based cART in the HAND. After the initiation of cART including stavudine, HIV individuals with cognitive impairment improve significantly as demonstrated by improved performance on a test of executive function [37]. Another prospective cohort study done in Uganda also confirmed that initiation of cART in HIVAD patients was associated with improvement in neurocognitive and functional performance in HIV individuals [38]. These results suggest that cART had good alternatives for the management of patients with HIVAD in areas with limited resources like SSA and providing appropriate treatment in patients with HIVAD to improve neurocognitive and functional performance in HIV individuals in SSA was important.

In low-income settings particularly in SSA, dementia is under-recognized, under-treated, or under-managed [26]. These factors make an evaluation, treatment, and research on dementia in these settings uniquely challenging, with specialist and culturally specific tools, and methods for assessment and monitoring of treatment required [28, 29]. Research has shown that the quality of care received by a person with dementia positively relates to a longer time spent being cared for at home, which is critical to the physical and mental health of the person with dementia [44]. Cognitive remediation intervention and social interaction using observational learning and participant modeling reduced disruptive behavior for persons with mild to moderate dementia living in the community. This reflects a general view that a lack of cognitive activity hastens cognitive decline. In SSA there is limited data on the pharmacologic management of dementia, however, on the non-pharmacologic intervention of dementia, especially on HAND, and those data were compiled by this systematic review, and most articles done in SSA indicated that CST and cART were the treatment modalities of dementia.

## 5. Limitations of the Systematic Review

There were some limitations to this systematic review. Only papers published in the English language were included in this systematic review, which due to the subject matter is a limitation. In addition, the systematic review included prospective cohort studies and the sample size of most studies was small, which may limit generalizability.

## 6. Conclusion

The results of this systematic review indicate that the majority of studies conclude that CST is feasible and clinically effective for the treatment of dementia in SSA. The improvements in cognitive function, behavioral symptoms, and quality of life after the intervention of CST were significant in physical, psychosocial, and environmental domains, and there is a change in the quality of life of people with dementia after the implementation of CST. Diagnosis and treatment of HIVAD have been difficult in SSA due to the late clinical presentation of HIV, advanced immunosuppression, and the high burden of often undiagnosed concurrent systemic infections. Early initiation of cART therapy in resource-limited settings has been associated with improved cognitive function in HIVAD.

## Figures and Tables

**Table 1:** Summary of RCTs and prospective cohort included in the analysis, 2021.

S. no	Study design	Subjects		Study purpose	Interventions	Duration of study	Dementia types/etiologies	Outcomes evaluated	Country	Authors, year	Conclusions of authors
		Control	Experimental/exposure								
1	RCT	642	9	Feasibility and clinical effectiveness of CST	Asses quality of life using the WHOQOL-BREF	7 weeks	Alzheimer's disease	Improvements in cognitive function and quality of life after CST intervention	Nigeria	Olakehinde et al., 2019	CST appears to be feasible in this setting.
2	RCT	466	34	Feasibility and clinical effectiveness of CST	Assess the changes in quality of life (QoL) by using WHOQOL-BREF	20 weeks	Alzheimer's disease	Change in QoL and cognition after CST intervention	Tanzania	Paddick et al., 2015	Intervention has the potential to be low-cost, sustainable, and adaptable to other settings across SSA.
3	RCT	—	94	The diagnostic accuracy of IHDS-HCSQ With HAND.	Administered the diagnostic accuracy of IHDS-HCSQ with HAND.	—	HAND	Cognitive screening tools might successfully discriminate The most severe form of HAND	South Africa	Gouse et al., 2017	IHDS-HCSQ combination offers a viable and quick way to screen people living with HIV for HAND. It can deliver excellent sensitivity and good specificity.
4	RCT	—	1036	Neurocognitive function at the first-line cART failure and change on the second-line therapy	Administered second-line therapy and test neurocognitive function at baseline, 48 and 96 weeks	96 weeks	HAND	Improved neurocognitive function after the administration of second-line therapy	5 SSA countries	Kambugu et al., 2016	Patients in SSA failing the first-line cART had low neurocognitive function test scores, but performance improved on the second-line cART.
5	Cohort	—	277∗	Identification and intervention for dementia in elderly Africans cognitive screen	Administered IDEA cognitive screen tool	—	Alzheimer's disease	Validation of the IDEA cognitive screen tool in SSA	Nigeria, Tanzania	Paddick et al., 2015	The IDEA cognitive screen performed well in these populations and should prove useful in screening for dementia and delirium in other areas of SSA
6	RCT	36	37	Efficacy and safety of minocycline in the management of HAND.	Receive 100 mg of minocycline orally every 12 hours for 24 weeks	24 weeks	HAND	Change in neurocognitive performance from baseline to 24 weeks	Uganda	Nakasujja et al., 2013	It did not improve HIV-associated cognitive impairment
7	Cohort	25	102∗	Benefits and risks of stavudine-based cART for HAND	Initiation of the stavudine-based cART	24 weeks	HAND	Improvement in verbal memory, motor and psychomotor speed, executive thinking, and verbal fluency	Uganda	Sacktor et al., 2009	After the initiation of cART, including stavudine, HIV individuals with cognitive impairment improve significantly as demonstrated by improved performance on a test of executive function
8	Cohort	—	23∗	Neuropsychological test and functional performance in HIV individuals after 3 and 6 months of cART	Initiation of cART		HAND	Neurocognitive at 3 and 6 months after initiation of cART	Uganda	Sacktor et al., 2006	cART can be associated with improvement in neurocognitive and functional performance in HIV individuals in SSA

∗Indicated the Cohort study design.

## Data Availability

Data supporting this research article are available from the corresponding author or first author upon reasonable request.

## Abbreviations

cART: combination anti-retroviral therapy
CST: Cognitive stimulation therapy
HIVAD: Human immunodeficiency virus associated dementia
HCSQ: HIV Cognitive Symptom Questionnaire
HIV: Human immunodeficiency virus
IDEA: Identification and intervention for dementia in elderly Africans
IHDS-HCSQ: International HIV Dementia Scale-HIV Cognitive Symptom Questionnaire
MeSH: Medical Subject Heading
RCT: Randomized control trials
SSA: Sub-Saharan Africa
WHOQOL-BREF: World Health Organization Quality of Life Instruments.
